# Transcriptome Analysis of Flounder (*Paralichthys olivaceus*) Gill in Response to Lymphocystis Disease Virus (LCDV) Infection: Novel Insights into Fish Defense Mechanisms

**DOI:** 10.3390/ijms19010160

**Published:** 2018-01-05

**Authors:** Ronghua Wu, Xiuzhen Sheng, Xiaoqian Tang, Jing Xing, Wenbin Zhan

**Affiliations:** 1 Laboratory of Pathology and Immunology of Aquatic Animals, Key Laboratory of Mariculture, Ministry of Education, Ocean University of China, 5 Yushan Road, Qingdao 266003, China; wurh2015@swu.edu.cn (R.W.); tangxq@ouc.edu.cn (X.T.); xingjing@ouc.edu.cn (J.X.); wbzhan@ouc.edu.cn (W.Z.); 2 Laboratory for Marine Fisheries Science and Food Production Processes, Qingdao National Laboratory for Marine Science and Technology, Qingdao 266071, China

**Keywords:** *Paralichthys olivaceus*, lymphocystis disease virus, transcriptome sequencing, differentially expressed genes

## Abstract

Lymphocystis disease virus (LCDV) infection may induce a variety of host gene expression changes associated with disease development; however, our understanding of the molecular mechanisms underlying host-virus interactions is limited. In this study, RNA sequencing (RNA-seq) was employed to investigate differentially expressed genes (DEGs) in the gill of the flounder (*Paralichthys olivaceus*) at one week post LCDV infection. Transcriptome sequencing of the gill with and without LCDV infection was performed using the Illumina HiSeq 2500 platform. In total, RNA-seq analysis generated 193,225,170 clean reads aligned with 106,293 unigenes. Among them, 1812 genes were up-regulated and 1626 genes were down-regulated after LCDV infection. The DEGs related to cellular process and metabolism occupied the dominant position involved in the LCDV infection. A further function analysis demonstrated that the genes related to inflammation, the ubiquitin-proteasome pathway, cell proliferation, apoptosis, tumor formation, and anti-viral defense showed a differential expression. Several DEGs including *β actin*, toll-like receptors, cytokine-related genes, antiviral related genes, and apoptosis related genes were involved in LCDV entry and immune response. In addition, RNA-seq data was validated by quantitative real-time PCR. For the first time, the comprehensive gene expression study provided valuable insights into the host-pathogen interaction between flounder and LCDV.

## 1. Introduction

Lymphocystis disease virus (LCDV), which belongs to the *lymphocystivirus* genus within *Iridoviridae* family and has a dsDNA genome, is the causative agent of lymphocystis disease, which has affected more than 140 marine and freshwater fish species worldwide, including flounder (*Paralichthys olivaceus*), an economically important fish in Asian countries such as Japan, Korea, and China [1], resulting in great economic losses [2,3,4,5]. The flounder suffering from lymphocystis disease is characterized by the appearance of tumor-like nodules consisting of hypertrophied lymphocystis cells on the skin, fins, gill, mouth, and even the internal organs, thus leading to loss of commercial value and sometimes causing death [1]. So far, great advances have been made with respect to the morphologic and genomic characterizations [6,7,8], and the pathology, epidemiology, and diagnosis methods of LCDV [9,10,11]. In addition, lymphocystis disease is partially controlled by antiviral molecules, vaccines, and immunostimulants that enhance the fish innate immune response and disease resistance [12,13,14]. However, the molecular mechanisms associated with LCDV pathogenesis and virus-host interactions are largely unknown due to the limited amount of available genomic information on flounder.

The mechanism of lymphocystis cell formation from the viewpoint of differentially expressed genes (DEGs) in the infected flounder fin has been investigated recently by using microarrays [15]; however, microarray technology has limited capacity to identify novel transcriptional profiles and quantify transcripts at a relatively low level [16]. Along with the rapid development of next generation sequencing, high-throughput RNA-sequencing (RNA-seq) technologies have become revolutionary tools for transcriptomics and genome characterization [17,18,19]. Rather than measuring relative gene expression, RNA-seq generates absolute information and is more sensitive to low-expressed transcripts [20], which makes it feasible to analyze the complexity of whole eukaryotic transcriptomes with less bias, a greater dynamic range, a lower frequency of false-positive signals, and higher reproducibility [21,22]. By using RNA-seq techniques, transcriptional responses of flounder to low temperature have been reported [23]. To our knowledge, no studies concerning the transcriptome analysis of flounder in response to LCDV infection using RNA-seq have been published.

The fish gill is one target organ of LCDV; it also plays an important role in the mucosal immune response as one of the mucosal barriers. In our previous research, LCDV copies were detected by quantitative real time PCR (qPCR) in flounder tissues at different time points post LCDV infection by intramuscular injection, and it was found that the copy number of LCDV in the gill increased from 12 h to 4 week in a time-dependent manner [24], while the detailed fish defense mechanisms for LCDV infection were not clear.

In this study, the gill from the LCDV-infected and non-infected control groups at one week post intramuscular injection [24] was used to prepare sequencing libraries, and the transcriptional sequence of flounder gill and a comparative analysis of transcriptome data between the two groups was performed using Illumina HiSeq 2500 sequencing platform. qRT-PCR was conducted to verify several randomly selected DEGs. The resultant data disclosed a large amount of novel gene information and showed significant expression changes in response to LCDV infection, which would promote a better understanding of pathogenesis and fish defense mechanisms to LCDV infection.

## 2. Results

### 2.1. RNA Quality Verification

The results of RNA (LCDV-G1, LCDV-G2; PBS-G1, PBS-G2) integrity detection were shown in Table 1. The absorbance ratio of RNA OD_260_/OD_280_ was less than 1.98 and greater than 1.85, and the brightness of the 28S band was about twice that of the 18S band. RNA integrity number (RIN) was about 9.0 in all samples.

### 2.2. Transcriptome Sequencing and Assembly

In total, there were 88,639,622 and 114,920,260 raw reads with an average length of 125 bp obtained from flounder gill of the controls (PBS-G1, PBS-G2) and LCDV-infected groups (LCDV-G1, LCDV-G2), respectively. A total of 86,643,614 and 106,581,556 clean reads were left after removing reads with adaptors, reads containing poly-N, and low-quality reads from raw data (Table 2).

**Figure 1:**
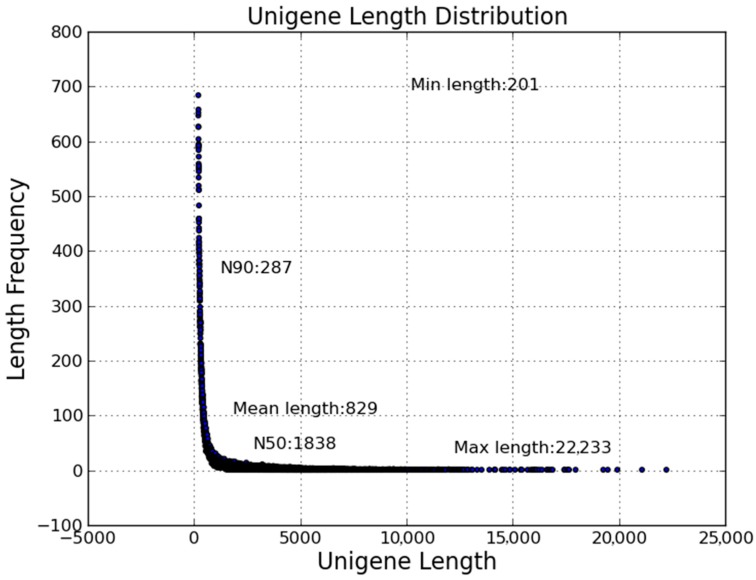
Length distribution of assembled unigenes. *X*-axis, unigene length; *Y*-axis, length frequency.

The clean reads of infected and control groups were combined and used to draw the transcriptome information of flounder gill. In total, 106,293 unigenes ranging from 201 bp to 22,333 bp, with a N50 length of 1838 bp, was generated from 193,225,170 combined clean reads (Figure 1). All the sequencing data were submitted to the NCBI database under the accession number SRP051613.

### 2.3. Functional Annotation of Unigenes Based on GO, KOG, and KEGG Analysis

Sequences of assembled unigenes were searched against seven different databases. Analyses showed that 24,922 unigenes (23.44%) had significant matches in the Nr database, 27,528 (25.89%) in the Nt database, and 21,572 (20.29%) in the Swiss-Prot database (Table 3). Totally, 36,537 unigenes (34.37%) were successfully annotated in at least one of the Nr, Nt, Swiss-Prot, KEGG, GO, KOG, and Pfam databases, with 8207 unigenes (7.72%) in all seven databases.

**Figure 2:**
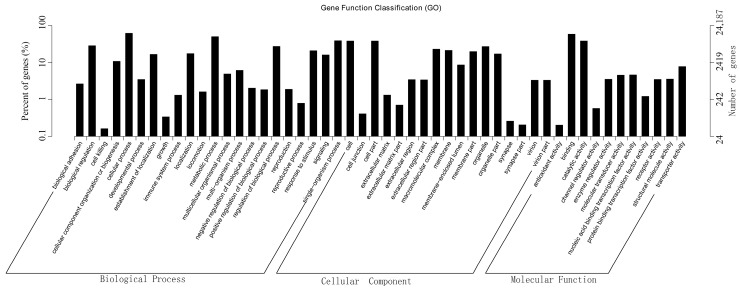
GO classification of assembled unigenes. *X*-axis, three major functional categories of GO terms: biological process, cellular component, and molecular function; *Y*-axis, terms with numbers/percents of unigenes in the major category.

For GO analysis, functions of the unigenes were classified with GO, an international standardized gene functional classification system that defined genes in three ontologies: molecular function, cellular component, and biological process. A total of 24,187 unigenes (22.75%) had GO annotation and were further assigned to 50 functional terms (Figure 2). In biological process category, genes involved in cellular process, metabolic process, and single-organism process were highly represented. Cellular component category mainly comprised terms of cell, cell part, and organelle. In molecular function category, genes related to binding, catalytic activity, and transporter activity were highly represented.

**Figure 3:**
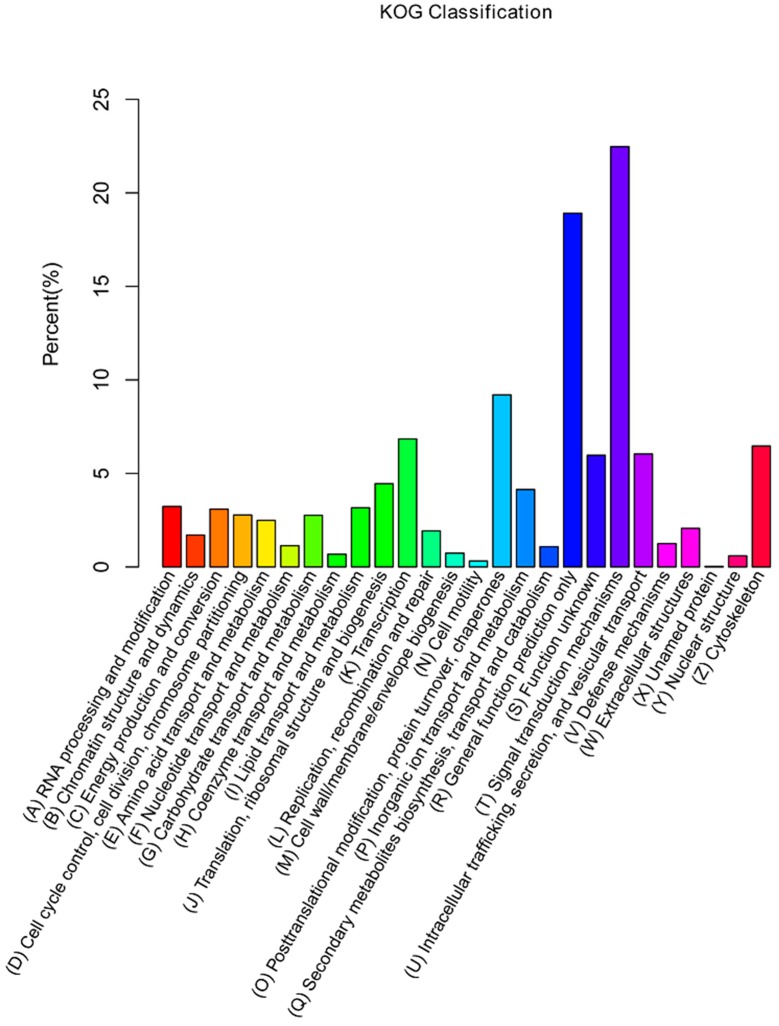
KOG classification of assembled unigenes. *X*-axis, the name of 26 groups in KOG; *Y*-axis, percentage of gene annotated in the group.

Additionally, unigenes were subjected to a search against the KOG database for functional prediction and classification. A total of 13,871 unigenes were assigned to KOG classification and divided into 26 specific categories (Figure 3). The predominant category was the cluster of signal transduction mechanisms (3117), followed by general functional prediction only (2624), post translational modification, protein turnover, chaperones (1276), transcription (949), and cytoskeleton (897). A few unigenes were assigned to nuclear structure (82) and cell motility (44).

**Figure 4:**
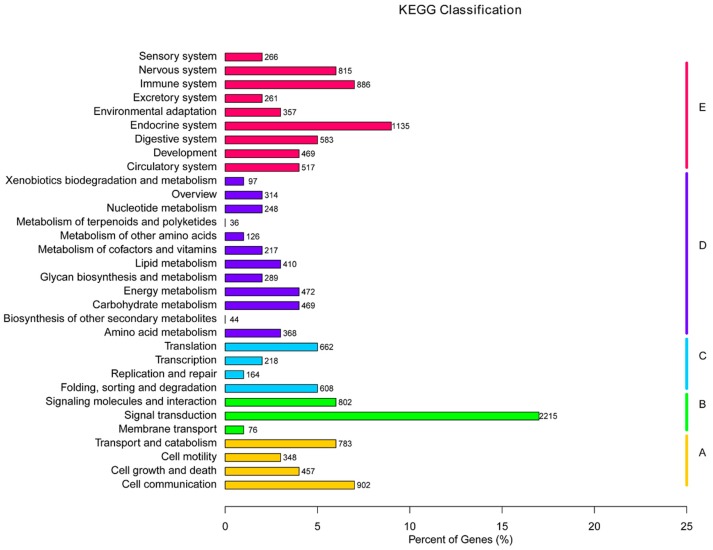
KEGG classification of the 23,468 unigenes. Five capital letters with the corresponding colored bars indicate five main categories. **A**: cellular processes; **B**: environmental information processing; **C**: genetic information processing; **D**: metabolism; **E**: organism systems.

Orientation of unigenes in metabolic pathways was conducted using the KEGG database, and 23,468 of the 36,537 unigenes were assigned to 31 pathway hierarchies containing 257 known KEGG pathways, which were divided into five branches with numbers/percentages of unigenes: cellular processes (2983, 8.16%), environmental information processing (5327, 14.58%), genetic information processing (1823, 4.99%), metabolism (4232, 11.58%), and organismal systems (9103, 24.91%) (Figure 4). Among the 31 KEGG pathways, the pathways involving homologs of the most unigenes were signal transduction (2215), followed by endocrine system (1135), cell communication (902), immune system (886), nervous system (815), and signaling molecules and interaction (802). No more than 50 unigenes were assigned to biosynthesis of other secondary metabolites and metabolism of terpenoids and polyketides.

### 2.4. Differential Gene Expression Induced by LCDV Infection

**Figure 5:**
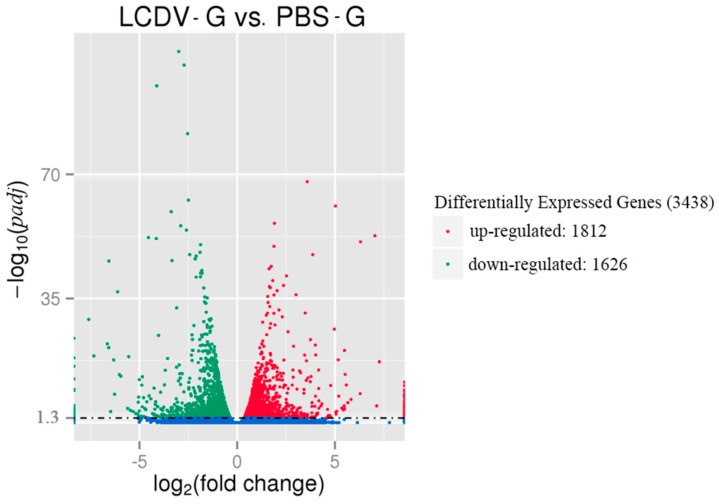
The ‘‘volcano plot’’ picture of DEGs between LCDV-infected and control groups. Red spots and green spots represented significantly up-regulated and down-regulated genes, respectively (*padj* < 0.05 and |log_2_ (fold change)| > 1). Blue spots: no difference in gene expression.

The DEGs between the LCDV-infected and control groups (LCDV-G vs. PBS-G) were identified as *padj* < 0.05. There were 3438 DEGs consisting of 1812 up-regulated and 1626 down-regulated unigenes in the LCDV-infected group when compared with the control group, as shown in the “volcano plot’’ picture (Figure 5). The randomly selected 30 of 1812 up-regulated and 1626 down-regulated DEGs were listed in Tables S1 and S2, respectively.

### 2.5. GO Classification of Differentially Expressed Genes (DEGs)

**Figure 6:**
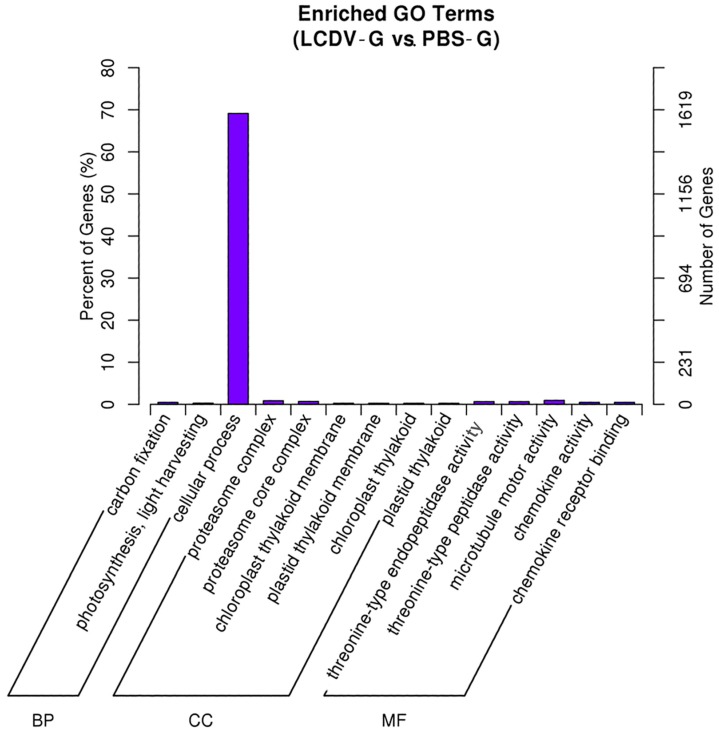
Enriched GO Terms of DEGs. *X*-axis, three main categories: biological process (BP), cellular component (CC), and molecular function (MF); *Y*-axis, number/percent DEGs of a term in the total annotated DEGs.

To understand the functions of DEGs, we mapped all the DEGs to terms in GO database. 2313 out of 3438 DEGs had a GO identity (ID) and were classified into three major functional categories (molecular function, cellular component, and biological process). GO analysis showed that genes related to term of cellular process were dominant (Figure 6). GO terms of chemokine activity, chemokine receptor binding, proteasome complex, and proteasome core complex, which were associated with inflammation, ubiquitin-mediated protein degradation, and apoptosis in cells, were also significantly enriched. All DEGs enriched in these terms were significantly up-regulated (Figure S1).

### 2.6. Pathway Classification Enrichment of DEGs

After mapping to the KEGG database, a total of 15 KEGG pathways involving 436 DEGs were significantly enriched (corrected *p* value < 0.05) in pathways categorized in four branches: genetic information processing, cellular processes, metabolism, and environmental information processing (Table 4), of which the DEGs enriched in metabolism were dominant. In details, eight pathways were divided into genetic information processing, such as proteasome, DNA replication, and mismatch repair; two pathways were divided into cellular processes, including p53 signaling pathway and cell cycle; four pathways were divided into metabolism, including amino sugar and nucleotide sugar metabolism, metabolic pathway, glutathione metabolism, and other types of *O*-glycan biosynthesis; only one pathway of tumor necrosis factor (TNF) signaling pathway was assigned to environmental information processing.

Particularly, among the 15 significantly enriched pathways, two pathways were involved in cell proliferation, including cell cycle and DNA replication, and most of the involved DEGs were up-regulated; three pathways (proteasome, p53 signaling pathway, and TNF signaling pathway) were related to apoptosis, and the majority of DEGs assigned to them were up-regulated. In addition, pathway of other types of *O*-glycan biosynthesis was involved in tumor formation.

### 2.7. Virus Entry- and Immune-Related DEGs in Response to LCDV Infection

Based on the functional annotation analysis, several DEGs were involved in virus entry and immune response after LCDV infection, including *β actin*, toll-like receptors (TLRs), cytokine-related genes, antiviral related genes, and apoptosis-related genes, which were partly shown in Table 5. At one week post LCDV infection, gene expression of *β actin*, *TLR3*, and *TLR14* were up-regulated. Cytokine-related DEGs were divided in five subcategories, including interferon (e.g., interferon γ and interferon regulatory factor 8), interleukin (e.g., interleukin 15 and interleukin-8 receptor), chemokine (e.g., CXC chemokine (Cys-X-Cys subfamily of chemokine. Members of this subfamily are characterized by two cysteines separated by a single amino acid) and CXC chemokine receptor 3), TNF (e.g., TNF ligand superfamily member 13B and TNF receptor-1), and granulocyte colony-stimulating factor. Several antiviral-related genes were also involved in the defense of LCDV infection, such as interferon-stimulated gene 15 (*ISG15*), complement component *C3*, and *Mx* and antimicrobial peptide NK-lysin-like. Some genes related to apoptosis showed different expression patterns: expression of perforin, natural killer enhancing factor, and granzyme II were up-regulated, while expression of *bcl-2* (B-cell lymphoma-2) was down-regulated in response to LCDV infection. Additionally, up-regulation of immunoglobulin D (IgD), and polymeric immunoglobulin receptor (pIgR), revealed the occurrence of adaptive immune response.

### 2.8. Validation of RNA-seq Data by qRT-PCR

**Figure 7:**
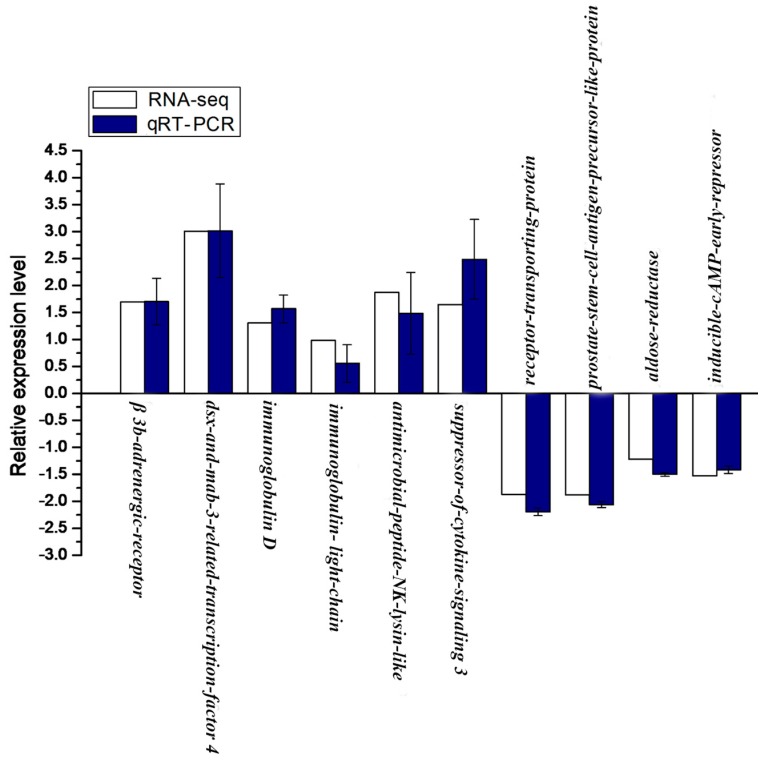
Validation of RNA-seq data by qRT-PCR. *Y*-axis, the relative expression level was expressed as log_2_ (fold change) in gene expression; *X*-axis, gene name. The relative expression of 10 genes, were detected by RT-qPCR (blue column) and compared with the results of RNA-seq (white column). Error bars represented standard deviation (SD).

The relative mRNA expression level of 10 genes, including 6 up-regulated and 4 down-regulated genes from statistical analysis of RNA-seq, were randomly selected and detected by qRT-PCR. The melting-curve analysis of qRT-PCR revealed a single product for all genes. As shown in Figure 7, 10 genes exhibited a concordant direction both in RNA-seq and qRT-PCR analysis. The correlation coefficient between RNA-seq and qRT-PCR results was 0.87, confirming the validation of RNA-seq data.

## 3. Discussion

The fish gill is an important tissue associated with immune responses, especially playing a crucial role in mucosal immunity as one of the mucosal barriers [25,26,27,28,29,30], and gill tissue is susceptible to LCDV infection [31]. Moreover, two putative cellular receptors responsible for LCDV infection have been identified in flounder gill cells [32,33,34]. Thus, the transcriptome sequencing of flounder gill will promote the understanding of LCDV-host interactions and identify abundant immune-related genes in response to LCDV infection.

The direct evidence of LCDV infection at one week post-intramuscular injection was provided in our previous research by calculating LCDV loads using qPCR [24]. In this study, RNA-seq was used to compare the differential transcription of the genes between the LCDV-infected and healthy flounder gill. The RNA-seq data from the gill with and without LCDV infection generated 193,225,170 clean reads aligned with 106,293 unigenes, which provided abundant data for the analysis of biological processes during LCDV invasion. Gene expression level was determined by calculating the number of unambiguous reads for each gene normalized using FPKM method [35], which obtained 3438 of differential transcription of genes including 1812 up-regulated and 1626 down-regulated genes. A recent study by using the oligo microarrays depicted 114 DEGs in the fin of flounder at one week post LCDV infection [15]. Therefore, RNA-seq could exhibit better performance on generating more novel transcripts than those traditional methods. Moreover, qRT-PCR results confirmed the reliability and accuracy of RNA-seq data available for the deep research of the complexity of the transcriptome.

To facilitate the global analysis of gene expression, the DEGs were assigned to different functional categories using GOseq R package [36]. In the present study, DEGs were significantly enriched and dominant in the term of cellular process, suggesting that the invasion of LCDV might interfere with the normal cellular process in order to obtain more cellular resources for synthesis of LCDV protein. According to the references, to gain prior transcription and translation of virus genes, many viruses possess strategies to disrupt the host normal cellular processes [37]; some viruses, such as infectious hematopoietic necrosis virus, can trigger a full scale inhibition of cellular host genes [38,39], and cellular resources available for the virus may be increased by host cell shutoff, leading to an increase of translation of viral proteins [39,40]. In LCDV-infected flounder gill, several up-regulated genes were significantly enriched in GO terms of chemokine activity, chemokine receptor binding, proteasome complex, and proteasome core complex, which were correlated with inflammation and apoptosis. It is now clearly known that chemokines and chemokine receptors play critical roles in inflammation, promoting the mobility of mononuclear cells throughout the body, activating the specific immune responses, and facilitating the pathogenesis of various diseases [41]. Proteasome complex plays a crucial role in ubiquitin-mediated protein degradation [42] and has been strongly implicated in apoptosis [43]. In this study, the enrichment of DEGs in proteasome complex suggested apoptosis due to LCDV disruption occurred in gill cells. Consistently, cell apoptosis were observed in vitro in flounder gill cell lines post LCDV infection [44,45].

To identify the biological pathways that were active in LCDV-infected gill, we mapped all the DEGs in the KEGG database. A large majority of the notably enriched KEGG pathways were associated with metabolism, in which most DEGs were enriched, suggesting that LCDV infection significantly perturbed the metabolism of flounder gill, as happened in the *Sogatella furcifera* in response to southern rice black-streaked dwarf virus [46]. Pathway of proteasome was significantly enriched, which was consistent with the GO enrichment analysis. Two significantly enriched pathways, p53 signaling pathway and TNF signaling pathway, were also apoptosis-related pathways [47,48], and the majority of DEGs assigned in them were up-regulated. During antiviral activities, the apoptotic cells might be replaced by inducing an excessive cell proliferation. Correspondingly, cell cycle and DNA replication, two cell proliferation-related pathways [49,50], were significantly enriched, and most DEGs involved in them were up-regulated, which might facilitate an excessive cell proliferation in the LCDV-infected gill cells. p53 signaling pathway can be activated when DNA damage happens, which plays an important role in DNA damage repair; it also exerts the role of inducing apoptosis when DNA damage repair fails [51], while the inactivation of p53 will potentially lead to tumor formation [52]. Moreover, it was reported that abnormal change of *O*-glycan was one of potential mechanisms for tumor immunological escape [53]. The present study found that the pathway of other types of *O*-glycan biosynthesis was also significantly enriched, which might be related to lymphocystis cell formation in LCDV-infected gill; further studies are worth conducting.

The first stage of virus replication cycle is attachment to the host cell surface; cellular receptors are the key molecular determinants regulating tissue tropism [54]. In the previous researches, a 27.8 kDa protein from flounder gill cells was identified as a receptor mediating LCDV entry and infection; mass spectrometry and western blotting analysis showed that the 27.8 kDa receptor protein had an association with β actin [32,33], and the expression of 27.8 kDa receptor protein was up-regulated post LCDV infection, indicating a positive correlation with efficient LCDV replication in the tissues of flounder and turbot [45,55]. In this study, the expression of β actin was up-regulated post LCDV infection; thus, more research is required to clarify the role of β actin in LCDV infection.

The TLRs can identify pathogens or products derived from pathogen and activate non-specific host defenses, such as inflammatory responses through induction of anti-microbial genes and inflammatory cytokines [56,57]. Additionally, there is accumulating evidence that TLRs can facilitate activation of specific immune responses [58,59]. In this study, *TLR3* and *TLR14* expression were up-regulated and closely related to LCDV infection. Interestingly, TLR3 has been implicated in RNA virus recognition, while a human DNA tumor virus up-regulating *TLR3* during primary infection is also reported [60]. In particular, a recent study shows that *TLR14* gene, which is unique to fish, is up-regulated in flounder post viral hemorrhagic septicemia virus infection [61].

Cytokine is one of the most important parts of innate immune system; it is the first line of defense against infectious disease. In this study, LCDV infection significantly regulated five members of cytokine-related DEGs, including interferon, interleukin, chemokine, TNF, and colony-stimulating factor; most of them were up-regulated except *ISG15*, *C-C motif chemokine 20 precursor*, *C-C motif chemokine 4-like*, *C-C chemokine receptor type 2-like*, *TNF receptor-1*, *and granulocyte colony-stimulating factor*. Of these cytokines, chemokine, interferon *γ*, and TNF, which were considered as pro-inflammatory cytokines [62], were up-regulated in LCDV-infected gill, while granulocyte colony-stimulating factor, which acted as an anti-inflammatory cytokine [63], was down-regulated post LCDV infection in flounder gill. In general, to prevent tissue damage resulted from an overactivation of immune cells, inflammation is strictly controlled [64]; therefore, the up-regulated expression of pro-inflammatory cytokines and down-regulated expression of anti-inflammatory cytokine might disturb the balance between pro- and anti-inflammation, leading to damage or cell apoptosis of flounder gill. Correspondingly, genes of perforin, natural killer enhancing factor, granzyme II, and *bcl-2*, which were related to cell apoptosis, exhibited differential expression in response to LCDV infection. *Bcl-2* could prevent apoptosis induced by perforin and granzyme B [65]; we deduced that the down-regulation of *bcl-2* and the up-regulation of perforin, natural killer enhancing factor, and granzyme II would facilitate the biological process of cell apoptosis induced by LCDV. Expression of interleukin or its analogue was also altered by LCDV infection. Interleukin-15 is not necessary for generating memory CD8 T cells, but it is essential for homeostatic proliferation to keep populations of memory cells over an extended period of time [66], so the up-regulation of interleukin-15 indicated that a similar function might exist in flounder. Interleukin-12, together with interferon gamma, plays a vital role in fighting viral infections [67,68]. In present study, interleukin-12 subunit beta-like was found to be up-regulated, revealing a potential role of interleukin-12 in anti-LCDV infection.

The non-specific immune system is triggered within minutes after pathogen invasion and bears responsibility for the infection resistance during the initial hours and days, while specific immunity needs at least 7–10 days to induce an appropriate cellular or humoral response [69]. At one week post LCDV infection, IgD and pIgR expression were up-regulated. It seemed that the injection could stimulate the immune response of IgD earlier than IgM, as the expression of IgD in almost all the tissues, including gill, was significantly elevated at one week after injection with bacterial pathogen *Flavo-bacterium columnare* G_4_ [70]. The function of fish IgD is unclear, yet it may play an important role in non-specific immunity. As shown in channel catfish *Ictalurus punctatus*, the secreted IgD can attach to basophils to trigger proinflammatory cytokines [71,72]. pIgR plays a dominant role in the mucosal immune system, the first line of specific immune defense, by mediating polymeric immunoglobulins (pIgs) such as polymeric IgM/IgT into fish mucosal secretions [73,74,75,76,77]. It is reported that the bacteria and viruses are able to up-regulated or down-regulated pIgR expression [78], and the up-regulation of pIgR expression can promote the efficient secretion of pIgs at mucosal surfaces and help the host clear the pathogens effectively [79]. However, it was strange that the IgM gene expression was not detected at one week after LCDV infection in this study. In addition, other DEGs induced by LCDV infection, such as ISG15, Mx and complement component C3, and antimicrobial peptide NK-lysin-like have been implicated in anti-viral defense [80,81]. However, one puzzling problem was that *ISG15* and *Mx* expression were down-regulated; this expression pattern might not benefit the anti-LCDV defense and was different from other host defenses against virus infections [82,83]. In a recent study, a high activation of *Mx* was detected in kidney of Senegalese sole (*Solea senegalensis*), which was infected by viral hemorrhagic septicemia virus [84]; possible reasons for the discrepancy might be because our sample was taken from gills rather than fish internal organs, or because different fish were challenged with different viruses, so further studies are needed.

In conclusion, we studied the transcriptome responses of flounder gill to LCDV infection, and the results were anticipated to explain the molecular basis of early response of host to LCDV infection. The invasion of LCDV interfered with the normal cellular process in order to obtain more cellular resources for synthesis of LCDV. DEGs were significantly enriched in pathways of cell cycle, DNA replication, proteasome, p53 signaling pathway, TNF signaling pathway, and other types of *O*-glycan biosynthesis, which were related to cell proliferation, apoptosis, and tumor formation. During virus-host interactions, some immune related- and antiviral genes were activated. Our comprehensive gene expression study not only described for the first time the entire host responses to LCDV infection in the early infection phase, but also provided new information for identification of novel genes in flounder.

## 4. Materials and Methods

### 4.1. Ethics Statement

Handling of flounder was approved by the Institutional Animal Care and Use Committee of Ocean University of China (Permit Number: 20111201; 27 March 2009). Fish were anesthetized with ethyl 3-amino-benzoate-methanesulfonic acid (MS222) before sacrificing, and handling and efforts were made to minimize suffering. All fish experiments strictly complied with the ARRIVE guidelines [85].

### 4.2. Experimental Fish and Virus Infection

Healthy flounders, 700–900 g in weight, were obtained from a fish farm in Qingdao of Shandong province, China [24]. The fish were tested negative for LCDV infection by PCR assay [32]. A total of 48 fish were cultured for 7 days before experiment as described previously [24]. The fish were maintained in aerated rearing tanks supplied with running seawater at 20 ± 1 °C; water temperature was kept constant for the experimental period.

The LCDV particles was collected from diseased flounder with lymphocystis nodules on the body surface, purified, and stored in our lab [4,33]. The concentration of LCDV was determined as described previously [44]. LCDV infection protocols were conducted as mentioned in our previous study [24]. Briefly, the flounders were randomly divided into two groups and lightly anesthetized in a solution of 30 mg MS222 per liter of water for ~3 min. The fish in group 1 were injected intramuscularly with 300 µL of the purified LCDV (diluted in PBS, 0.1 mg per fish). Gill tissue was sampled from 6 individuals at one week post LCDV injection and every three flounder gills were mixed together, defined as LCDV-G1 and LCDV-G2, respectively. The flounders in group 2 were intramuscularly injected with equal volume of PBS as negative control and gill tissues were sampled in parallel, which were defined as PBS-G1 and PBS-G2, respectively.

### 4.3. RNA Isolation and Quality Verification

Gill tissue samples from LCDV infection group (LCDV-G1, LCDV-G2) and control group (PBS-G1, PBS-G2) were subjected to RNA isolation and transcriptome analysis. The samples were isolated with Trizol reagent (Invitrogen, Carlsbad, CA, USA) following the manufacturer’s instruction. RNase-free DNase (Promega RQ1 DNase I, Promega, Madison, WI, USA) was used to remove any genomic DNA contamination by incubating for 30 min at 37 °C. RNA degradation was detected in 1% agarose gels. RNA purity was detected through a Nano Photometer spectrophotometer (IMPLEN, Westlake Village, CA, USA). RNA concentration was determined using Qubit RNA Assay Kit in Qubit 2.0 Fluorometer (Life Technologies, Camarillo, CA, USA). RNA integrity was measured by using the RNA Nano 6000 Assay Kit of the Agilent Bioanalyzer 2100 system (Agilent Technologies, Santa Clara, CA, USA). RNA from each mixed sample was divided into two parts: one for transcriptome sequencing and the remaining part for quantitative real-time PCR to validate the repeatability and reproducibility of DEGs data obtained from RNA sequencing.

### 4.4. Library Preparation for Transcriptome Sequencing

The protocols of library preparation for transcriptome sequencing were provided by Beijing Novogene Biotechnology Co., Ltd. in China. By using NEBNext Ultra RNA Library Prep Kit (NEB, Ipswich, MA, USA), sequencing libraries were prepared according to manufacturer’s recommendations, together with the addition of index codes to attribute sequence to corresponding sample. In brief, purification of mRNA from a total amount of 3 μg RNA obtained from LCDV challenge or control group was performed by using poly-T oligo-attached magnetic beads. The process of fragmentation was carried out using divalent cations at high temperature in NEBNext First Strand Synthesis Reaction Buffer (5×) (NEB). The resultant fragments were subjected to synthesizing first- and second-strand cDNA. For the first strand cDNA, it was synthesized by using random hexamer primer and M-MuLV Reverse Transcriptase (RNase H^−^). For the second strand cDNA, its synthesis was subsequently carried out using DNA polymerase I and RNase H. NEBNext Adaptor with hairpin loop structure were ligated to prepare for hybridization after adenylation of 3′ ends of DNA fragments. To select cDNA fragments of 150~200 bp in length, the library fragments were purified by employing AMPure XP system (Beckman Coulter, Beverly, MA, USA). Subsequently, a total of 3 μL USER Enzyme (NEB) was added in size-selected, adaptor-ligated cDNA for 15 min at 37 °C, then subjected to 5 min at 95 °C before PCR. Afterwards, PCR reaction was carried out with universal PCR primers, Phusion High-Fidelity DNA polymerase, and index primer. In the end, PCR products were subjected to purification, and library quality was evaluated on the Agilent Bioanalyzer 2100 system (Agilent). All the library preparations were subjected to sequencing on an Illumina HiSeq 2500 platform (Illumina, San Diego, CA, USA), and 125 bp in length of paired-end reads were produced.

### 4.5. TranScriptome Assembly and Annotation

The protocols of transcriptome assembly and annotation were provided by Beijing Novogene Biotechnology Co., Ltd. in China. Briefly, to get high-quality clean reads, raw reads of FASTQ format were firstly handled through in-house Perl scripts. In this procedure, clean reads were acquired by removing reads containing adapter, low quality reads, and reads containing poly-N from raw data. At the same time, the calculation of Q20, GC-content, and sequence duplication level were based on the high-quality clean reads. Transcriptome assembly was achieved using Trinity [86].

Gene function was annotated according to the seven databases: Nt (NCBI non-redundant nucleotide sequences), Nr (NCBI non-redundant protein sequences), KOG/COG (Clusters of Orthologous Groups of proteins), Pfam (Protein family), Swiss-Prot (A manually annotated and reviewed protein sequence database), GO (Gene Ontology), and KO (KEGG Ortholog database) using BLAST with a cutoff *E*-value of 10^−5^.

### 4.6. Quantification of Gene Expression Levels and Differential Expression Analysis

Gene expression levels of the samples were estimated by RSEM (RNA-Seq by Expectation Maximization, rsem-1.2.0), a user-friendly software package for quantifying gene abundances from paired-end RNA-Seq data [87]. Clean data were matched with the assembled transcriptome. Readcount of each unigene was derived from the mapping results and then normalized to FPKM (expected number of Fragments Per Kilobase of transcript sequence per Millions base pairs sequenced); one method was used to accurately assess gene expression level [35].

Differential expression analysis of genes across samples with biological replicates was performed using the package DEGseq [88]. DEGSeq provided statistical method for deciding differential expression genes; the resultant *p*-values were adjusted employing the reported methods for limiting the false discovery rate [89]. Genes with an adjusted *p*-value (*padj*) < 0.05 and log_2_ (fold change) of 1 were set as the threshold for significantly differential expression.

### 4.7. GO and KEGG Enrichment Analysis

GO enrichment analysis of the DEGs was carried out by the GOseq R packages based on Wallenius non-central hyper-geometric distribution [35], in which gene length bias in DEGs was adjusted.

According to the KEGG database resources, analysis of pathway enrichment could further seek out the notably enriched signal transduction pathways or metabolic pathways. KOBAS software (v2.0) was employed to detect the statistical enrichment of DEGs in KEGG pathways [90].

### 4.8. Quantitative Real-Time PCR 

To validate the repeatability and reproducibility of DEGs data obtained from RNA sequencing in the gill of flounder, qRT-PCR was implemented on 10 randomly selected DEGs using the same RNA as RNA-seq. Primers (Table S3) were designed according to Illumina sequencing data by using Primer Premier 5 (Premier Biosoft, Palo Alto, CA, USA). Reversed cDNA was synthesized using the PrimeScript RT reagent Kit with gDNA Eraser (Takara, Dalian, China). The reaction was carried out in a total volume of 20 μL, containing 10 μL SYBR Green Master Mix (Takara), 50 ng diluted cDNA mix, 0.6 μL each primer (10 mM), and 7.8 μL RNase-free water. The qRT-PCR amplifications were conducted with a Roche LightCycler 480 Real-Time PCR system (Roche, Basel, Switzerland) using SYBR Green Master Mix (Takara) according to the manufacturer’s instructions. The amplification was run at 95 °C for 5 min, followed by 40 cycles of 95 °C for 10 s, 58 °C for 20 s for annealing, and 72 °C for 30 s for extension. 18S RNA was used as an internal control to normalize the expression level, and all experiments were conducted in triplicate. After the end of PCR reactions, the genes expression level was determined according to the comparative CT method (2^−∆∆*C*^_T_ method) [91]. All data were expressed as means ± standard deviation. The statistical analysis was performed using the statistical software SPSS 17.0 (IBM Corp., Armonk, NY, USA).

## Figures and Tables

**Table 1:** The detection results of RNA from Agilent 2100.

Sample Name	OD_260_/OD_280_	28S/18S RNA Ratio	RIN Value
PBS-G1	1.853 ± 0.086	1.7 ± 0.3	8.9 ± 0.3
PBS-G2	1.887 ± 0.104	1.8 ± 0.2	8.7 ± 0.2
LCDV-G1	1.971 ± 0.018	2.1 ± 0.1	9.1 ± 0.1
LCDV-G2	1.865 ± 0.117	1.9 ± 0.2	9.3 ± 0.2

PBS, phosphate buffer solution; LCDV, lymphocystis disease virus; RIN, RNA integrity number.

**Table 2:** Summary of sequences analysis.

Category	PBS-G1	PBS-G2	LCDV-G1	LCDV-G2	Summary
Raw reads	47,382,188	41,257,434	55,497,824	59,422,436	203,559,882
Clean reads	46,280,260	40,363,354	51,413,710	55,167,846	193,225,170
Q20 (%)	93.12–95.21	93.13–95.29	92.05–96.24	91.72–96.35	
GC (%)	47.69–47.71	47.51–47.53	47.41–47.55	47.61–47.76	
Total mapped	39,092,630 (84.47%)	34,395,222 (85.21%)	43,059,442 (83.75%)	46,511,408 (84.31%)	

Q20: The percentage of bases with a Phred value >20.

**Table 3:** BLAST analysis of non-redundant unigenes against seven different databases.

Annotation in Database	Number of Unigenes	Percentage (%)
Annotated in Nr	24,922	23.44
Annotated in Nt	27,528	25.89
Annotated in **KEGG Ortholog**	12,881	12.11
Annotated in Swiss-Prot	21,572	20.29
Annotated in Pfam	23,245	21.86
Annotated in GO	24,187	22.75
Annotated in KOG	13,871	13.04
Annotated in all databases	8207	7.72
Annotated in at least one database	36,537	34.37
Total unigenes	106,293	100

**Table 4:** The 15 significant enrichment of pathways for differentially expressed genes (DEGs). The pathway ID was obtained from the KEGG database. Pathways with a corrected *p* value < 0.05 were significantly enriched.

Pathways	Pathways ID	Branch in the KEGG Database	Up-/Down-Regulated Genes
Proteasome	ko03050	genetic information processing	25/0
Base excision repair	ko03410	genetic information processing	9/1
Homologous recombination	ko03440	genetic information processing	7/2
DNA replication	ko03030	genetic information processing	12/0
Mismatch repair	ko03430	genetic information processing	7/0
Spliceosome	ko03040	genetic information processing	22/3
Aminoacyl-tRNA biosynthesis	ko00970	genetic information processing	10/0
Nucleotide excision repair	ko03420	genetic information processing	10/0
Metabolic pathways	ko01100	metabolism	128/77
Glutathione metabolism	ko00480	metabolism	11/3
Other types of *O*-glycan biosynthesis	ko00514	metabolism	6/4
Amino sugar and nucleotide sugar metabolism	ko00520	metabolism	9/7
p53 signaling pathway	ko04115	cellular processes	15/8
Cell cycle	ko04110	cellular processes	27/8
Tumor necrosis factor (TNF) signaling pathway	ko04668	environmental information processing	16/9

**Table 5:** Expression of virus-entry and immune-related DEGs in gill tissues at one week post LCDV infection.

Gene Name	Expression after Infection	Expression in Control	Ratio of Expression (Infection vs. Control)
*β actin*	12,056	7655	1.575
*TLR3*	38	4	9.500
*TLR14*	409	1259	0.325
*interferon regulatory factor 2*	286	138	2.072
*interferon regulatory factor*	15,252	9252	1.649
*interferon γ receptor α chain*	5521	3731	1.480
*interferon γ*	145	68	2.132
*interferon regulatory factor 8*	355	223	1.592
*interleukin-8 receptor*	217	49	4.429
*interleukin-12 subunit β-like*	564	315	1.790
*Interleukin-15*	413	242	1.707
*chemokine XC receptor 1-like*	589	216	2.727
*CC chemokine*	124	24	5.167
*C-C motif chemokine 3 precursor*	218	85	2.565
*CXC chemokine receptor 4*	4244	2835	1.497
*CXC chemokine receptor 3*	1009	634	1.591
*C-C motif chemokine 4 precursor*	284	158	1.797
*CXC chemokine*	1393	979	1.423
*chemokine XC receptor 1-like*	787	537	1.466
*C-X-C chemokine receptor type 4 protein*	166	90	1.844
*C-C motif chemokine 20 precursor*	111	361	0.307
*C-C motif chemokine 4-like*	26	115	0.226
*C-C chemokine receptor type 2-like*	82	207	0.396
*TNF ligand superfamily member 13B*	883	405	2.180
*TNF ligand superfamily member 6-like*	553	305	1.813
*TNF alpha-induced protein 2-like*	4776	2516	1.898
*TNF ligand superfamily member 14-like*	666	452	1.473
*TNF receptor-1*	1186	3661	0.324
*TNF ligand superfamily member 13B*	883	405	2.180
*TNF ligand superfamily member 6-like*	553	305	1.813
*TNF ligand superfamily member 14-like*	666	452	1.473
*granulocyte colony-stimulating factor*	21	129	0.163
*perforin*	2053	1136	1.807
*natural killer enhancing factor*	1209	628	1.925
*bcl-2*	402	641	0.627
*granzyme II*	3392	1213	2.796
*IgD*	47,080	19,068	2.469
*pIgR*	202	123	1.642
*ISG 15*	2199	3851	0.571
*Mx*	932	2203	0.423
*antimicrobial peptide NK-lysin-like*	1361	372	3.659
*complement factor D*	16,310	8844	1.844
*complement component C3*	123	61	2.016
*complement component C6-like*	197	57	3.456
*ubiquitin-conjugating enzyme E2 A*	352	221	1.593
*ubiquitin-conjugating enzyme E2 C-like*	340	142	2.394
*E3 ubiquitin-protein ligase CHFR-like*	2191	1409	1.555
*E3 ubiquitin-protein ligase RING2-like*	1023	701	1.459

## Supplementary Materials

The following are available online at www.mdpi.com/1422-0067/19/1/160/s1.
