# Supplementary material for: Transcriptome Analysis of Flounder (Paralichthys olivaceus) Gill in Response to Lymphocystis Disease Virus (LCDV) Infection: Novel Insights into Fish Defense Mechanisms
Source: Int J Mol Sci. 2018 Jan 5;19(1):160. doi: 10.3390/ijms19010160 (PMC5796109; doi:10.3390/ijms19010160)
Supplement: Supplementary file 1 [file ijms-19-00160-s001.pdf]

Supplementary Materials: Transcriptome Analysis of Flounder (*Paralichthys olivaceus*) Gill in Response to Lymphocystis Disease Virus (LCDV) Infection: Novel Insights into Fish Defense Mechanisms

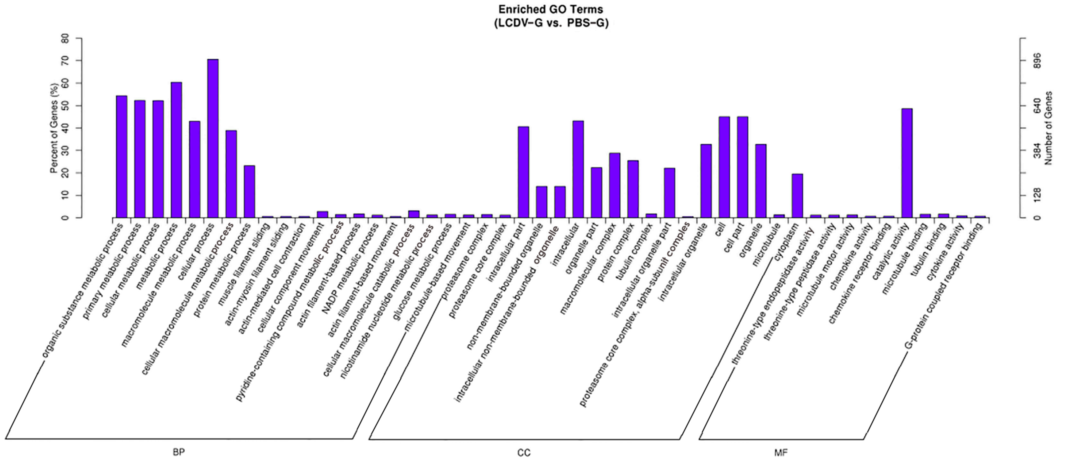

Figure. S1. Enriched GO Terms of up-regulated DEGs. *X*-axis, three main categories: BP (biological process), CC (cellular component) and MF (molecular function); *Y*-axis, numbers/percents DEGs of a term in the total annotated DEGs.

Table S1. The thirty up-regulated unigenes in flounder gill post LCDV infection.

Gene name	Species	Expression in control	Expression in infection	Corrected *p* value
goose-type lysozyme	*Paralichthys olivaceus*	10244	31785	$4.33E^{-44}$
suppressor of cytokine signaling 3	*Scophthalmus maximus*	1466	4593	$4.45E^{-39}$
granzyme II	*Paralichthys olivaceus*	1213	3392	$5.61E^{-31}$
immunoglobulin D	*Paralichthys olivaceus*	19068	47080	$4.69E^{-29}$
Amine sulfotransferase	*Anoplopoma fimbria*	1404	10381	$2.90E^{-22}$
serotransferrin precursor	*Anoplopoma fimbria*	29	462	$8.02E^{-20}$
immunoglobulin light chain	*Epinephelus coioides*	10827	21346	$2.93E^{-16}$
hepcidin precursor type I	*Paralichthys olivaceus*	141	635	$7.83E^{-13}$
complement factor D	*Paralichthys olivaceus*	8844	16310	$1.07E^{-12}$
novel immune-type receptor 5	*Dicentrarchus labrax*	214	551	$1.55E^{-11}$
Serine/threonine-protein kinase 6	*Dicentrarchus labrax*	86	247	$6.41E^{-10}$
perforin	*Paralichthys olivaceus*	1136	2053	$1.38E^{-09}$
Adenosine receptor A1	*Dicentrarchus labrax*	93	244	$3.16E^{-08}$
interleukin-8 receptor	*Paralichthys olivaceus*	49	217	$6.18E^{-08}$
Integrin alpha-M	*Dicentrarchus labrax*	2416	3876	$4.96E^{-07}$
C-C motif chemokine 3 precursor	*Anoplopoma fimbria*	85	218	$5.17E^{-07}$
Proteasome subunit alpha type-4	*Anoplopoma fimbria*	1097	1779	$2.38E^{-06}$
natural killer enhancing factor	*Paralichthys olivaceus*	628	1209	$4.63E^{-06}$
interferon regulatory factor	*Paralichthys olivaceus*	9252	15252	$1.44E^{-05}$
proteasome activator subunit 2	*Paralichthys olivaceus*	7202	10945	$4.00E^{-05}$
CXC chemokine receptor 4	*Scophthalmus maximus*	2835	4244	$4.34E^{-05}$
CXC chemokine receptor 3	*Scophthalmus maximus*	634	1009	$5.97E^{-05}$
toll-like receptor 3	*Paralichthys olivaceus*	4	38	$9.31E^{-05}$
Proteasome assembly chaperone 3	*Anoplopoma fimbria*	465	744	$1.86E^{-04}$
interleukin 15	*Anoplopoma fimbria*	242	413	$4.32E^{-04}$
fish-egg lectin	*Paralichthys olivaceus*	76814	107283	$6.58E^{-04}$

C-C motif chemokine 4 precursor	*Paralichthys olivaceus*	158	284	8.04E^{-04}
interferon γ	*Paralichthys olivaceus*	68	145	1.14E^{-03}
CXC chemokine	*Scophthalmus maximus*	979	1393	3.71E^{-03}
interferon regulatory factor 8	*Paralichthys olivaceus*	223	355	4.79E^{-03}

Table S2. The thirty down-regulated unigenes in gill post LCDV infection.

Gene name	Species	Expression in control	Expression in infection	Corrected *p* value
Apolipoprotein D	*Dicentrarchus labrax*	3134	1208	1.34E^{-26}
tumor necrosis factor receptor-1	*Paralichthys olivaceus*	3661	1186	1.59E^{-22}
inducible cAMP early repressor	*Oreochromis niloticus*	4184	1457	2.07E^{-18}
epigen	*Epinephelus bruneus*	3122	1458	3.62E^{-17}
C-C motif chemokine 20 precursor	*Epinephelus bruneus*	361	111	9.70E^{-16}
proteinase-activated receptor-2b	*Salmo salar*	4228	2370	1.95E^{-10}
B-cell translocation protein 1	*Lates calcarifer*	22567	12999	2.07E^{-10}
Rho-class glutathione-S-transferase	*Paralichthys olivaceus*	36043	17126	2.58E^{-10}
MHC class II antigen β domain	*Acipenser baerii*	6521	3765	6.67E^{-10}
toll like receptor 14	*Paralichthys olivaceus*	1259	409	1.05E^{-08}
Monocarboxylate transporter 6	*Dicentrarchus labrax*	1303	745	1.25E^{-07}
stromelysin-2 precursor	*Danio rerio*	262	108	1.56E^{-07}
reverse transcriptase	*Oryzias latipes*	173	63	4.38E^{-07}
T cell receptor γ chain V-J-C1	*Paralichthys olivaceus*	1228	715	7.41E^{-07}
iodothyronine deiodinase type I	*Paralichthys olivaceus*	588	293	2.17E^{-05}
Interleukin-17 receptor B	*Pteropus alecto*	285	143	3.23E^{-05}
interferon-inducible protein 56	*Larimichthys crocea*	8310	4563	4.46E^{-05}
Rhesus-associated glycoprotein	*Gasterosteus aculeatus*	2403	1270	7.74E^{-05}
interferon-stimulated gene 15	*Paralichthys olivaceus*	3851	2199	9.33E^{-05}
cytochrome b5 type A	*Epinephelus bruneus*	456	262	1.15E^{-04}
alpha2 a2 adrenergic receptor	*Takifugu rubripes*	201	73	1.48E^{-04}
granulocyte colony-stimulating factor	*Paralichthys olivaceus*	129	21	1.71E^{-04}
interleukin 17A/F	*Scophthalmus maximus*	27	2	3.08E^{-04}
cystatin C	*Scophthalmus maximus*	4611	3204	3.30E^{-04}
neurolin-b precursor	*Takifugu rubripes*	211	106	3.41E^{-04}

lipoprotein lipase	*Paralichthys olivaceus*	1357	560	4.58E^{-04}
selenoprotein U	*Paralichthys olivaceus*	571	353	4.98E^{-04}
hypoxia-inducible factor 1 alpha	*Platichthys flesus*	586	364	6.20E^{-04}
glutathione S-transferase	*Pleuronectes platessa*	330	194	1.28E^{-03}
heat shock protein HSP 90 alpha	*Platichthys flesus*	336	190	3.44E^{-03}

Table S3. Genes and specific primers used for real-time polymerase chain reaction.

Name	Amplicon length (bp)	Primers sequence (5′ to 3′)
18S RNA	107	GGTCTGTGATGCCCTTAGATGTC; AGTGGGGTTCAGCGGGTTAC
β-3b-adrenergic-receptor	116	GCAACACAGCAGGTCAAGC; GCACATACAGAGGGCAGGAT
dsx-and-mab-3-related-transcription-factor 4)	198	AGAAGCGGGACACACTGGA; GGGAGAAGGCGGATTTGT
Immunoglobulin D	100	ATGCAAGGCTCAACACAACA; GGGAATCTCCACATCAATGG
immunoglobulin-light-chain	125	GAGAAGGACGGCCACTACAG; TCCAGGGTTTGAGTGACAGG
antimicrobial-peptide-NK-lysin-like	169	GGGTTCTCAGCAAACACAGC; ACAGAGGGTTCCACATCCAC
suppressor-of-cytokine-signaling 3	101	TCGGAGAAACACTGAAGGTG; AAGAGGTGGAGACGAACAGG
receptor-transporting-protein	145	ACCGAGTGATGGTGGTCTTC; ATGAGGATGGTGATGTGCTC
prostate-stem-cell-antigen-precursor-like-protein	153	CCCAGAAATCCAATCCCAAT; AGGAGCCACAGCCCTTCAT
aldose-reductase	125	AGTCTCTGCTCGTCCTCTGG; GCTTTCACTGCTTCTGTCACC
inducible-cAMP-early-repressor	117	TGCAGAGAGAGAGAGCAGAGAG; GCAGCAGAACACCTCAATGT
